# Acupuncture for Improving Cognitive Impairment After Stroke: A Meta-Analysis of Randomized Controlled Trials

**DOI:** 10.3389/fpsyg.2020.549265

**Published:** 2020-11-30

**Authors:** Liang Zhou, Yao Wang, Jun Qiao, Qing Mei Wang, Xun Luo

**Affiliations:** ^1^Department of Reproductive Medicine, Peking University Shenzhen Hospital, Shenzhen, China; ^2^Department of Rehabilitation Medicine, Dapeng New District Nan'ao People's Hospital, Shenzhen, China; ^3^The Second Rehabilitation Hospital of Shanghai, Shanghai, China; ^4^Stroke Biological Recovery Laboratory, Spaulding Rehabilitation Hospital, The Teaching Affiliate of Harvard Medical School, Boston, MA, United States; ^5^Kerry Rehabilitation Medicine Research Institute, Shenzhen, China

**Keywords:** MMSE, cognitive impairment, electroacupuncture, acupuncture, post stroke

## Abstract

**Objective:** This meta-analysis evaluated the efficacy of acupuncture in improving cognitive impairment of post-stroke patients.

**Design:** Randomized controlled trials (RCTs) investigating the effects of acupuncture compared with no treatment or sham acupuncture on post-stroke cognitive impairment (PSCI) before December 2019 were identified from databases (PubMed, EMBASE, Ovid library, Cochrane Library, Chinese National Knowledge Infrastructure, VIP Chinese Periodical Database, Wanfang Database, and SinoMed). The literature searching and data extracting were independently performed by two investigators. Study quality was assessed using the Cochrane Handbook for Systematic Reviews of Interventions. Meta-analyses were performed for the eligible RCTs with Revman 5.3 software.

**Results:** Thirty-seven RCTs (2,869 patients) were included in this meta-analysis. Merged Random-effects estimates of the gain of MMSE (Mini-Mental State Examination) or MoCA (Montreal Cognitive Assessment) were calculated for the comparison of acupuncture with no acupuncture or sham acupuncture. Following 2–8 weeks of intervention with acupuncture, pooled results demonstrated significant effects of acupuncture in improving PSCI assessed by MMSE (MD [95% CI] = 2.88 [2.09, 3.66], *p* < 0.00001) or MoCA (MD [95% CI] = 2.66 [1.95, 3.37], *p* < 0.00001).

**Conclusion:** The results suggest that acupuncture was effective in improving PSCI and supported the needs of more rigorous design with large-scale randomized clinical trials to determine its therapeutic benefits.

## Introduction

Stroke is a disease that causes high rates of mortality and disability worldwide (Wu et al., 2010). Cognitive impairment is a frequent condition after stroke (Tatemichi et al., 1994; Patel et al., 2003), and its prevalence ranges from 17 to 92% (Pasi et al., 2012). Cognitive rehabilitation could enhance the quality of life for post-stroke patients, which included a comprehensive cognitive improvement program treating cognitive dysfunction involving disorientation, sensory disorders, attention disorders, executive function disorders, and memory disorders (Berrol, 1990; Choi and Twamley, 2013). The clinical depression is characterized by behavioral, cognitive, and emotional features (Merriman et al., 2019). Cognitive performance is associated with symptoms of depression (Nakling et al., 2017), and early cognitive impairment after stroke predicts long-term depressive symptoms in patients (Nys et al., 2006).

Acupuncture therapy has been used widely to promote motor recovery after stroke (Hu et al., 1993; Lee et al., 2003). Because of its low cost with low adverse events, acupuncture has also been used to improve the cognitive function of stroke patients, mostly in China, and it is receiving increasing attention among western countries (Johansson et al., 1993; NIH consensus conference, 1998). A considerable number of clinical trials showed the potential role of acupuncture as a promising treatment for post-stroke cognition impairment, but some trials suggest that acupuncture does not affect post-stroke cognitive impairment (PSCI) (Guo et al., 2007). The conflicting results may be caused by a small sample size of the trials and a flaw of study design.

Two systematic reviews (Liu et al., 2014; Wang, 2017) were performed; however, the studies were limited by small sample size. Liu et al. (2014) reported the meta-analysis results of 21 trials from 2006 to 2012; however, those trials had 12 different methods to evaluate cognitive function. Therefore, the largest dataset had only 116 patients from four studies [with Mini-Mental State Examination (MMSE) as the outcome measure]. In Wang's systematic review, only 15 studies with 1,085 subjects from 2008 to 2016 were included.

Since the last systematic review, many more clinical trials of acupuncture for post-stroke impairment were conducted; however, all of these clinical trials were limited by a small sample size or inconsistent selection criteria for the assessment of cognitive function. Therefore, with the further increased randomized controlled trials (RCT) evidence, there is a strong need to perform a systematic review to evaluate the therapeutic effect of acupuncture to treat PSCI.

In this study, we hypothesize that acupuncture is effective to improve cognitive function after stroke as compared to sham or no acupuncture. This systematic review and meta-analysis aimed to validate the efficacy of acupuncture in treatment for PSCI with MMSE or Montreal Cognitive Assessment (MoCA), which are the most generally used assessment tools for cognitive impairment (Foreman et al., 1996; Nasreddine et al., 2005).

## Materials and Methods

### Inclusion Criteria and Exclusion Criteria

The inclusion criteria were the following: (1) type of studies: only randomized controlled trials (RCTs) of acupuncture for PSCI in English or Chinese language which were published before December 2019 were included; (2) type of participants: post-stroke patients (over 18 years old) with PSCI were included without restriction on gender, race, or nation; (3) type of interventions: the RCTs that used traditional acupuncture or electroacupuncture to treat PSCI were included; (4) outcome measurements: the outcome was assessed by MMSE or MoCA; and (5) type of comparators: the comparative interventions could be sham acupuncture or conventional treatment with rehabilitation. A RCT was included if acupuncture was used at acupoints as the sole treatment or as an adjunct to other treatments for cognition impairment after stroke. If studies included three or over three groups with only one group receiving acupuncture, and there is a control group without receiving acupuncture treated consistent with the acupuncture group, the data of acupuncture group, and control group were chosen for this study. If studies included three or over three groups with two or over two groups receiving acupuncture, a routine acupuncture group was chosen as the experiment group, and the group not receiving any acupuncture treated consistent with the acupuncture group was chosen as a control group.

The exclusion criteria were the following: (1) cognition impairment caused by other diseases except for stroke; (2) studies without a control group (control group treated consistent with the acupuncture group except receiving acupuncture); (3) studies compared different types of acupuncture; (4) studies compared the effect of acupuncture with a drug; (5) studies adopted complex treatment without specifying the sole effects of acupuncture; (6) cognition outcome measured by another assessing system except for MMSE or MoCA; (7) studies without standardized indices of curative effect or detailed results of treatment will be excluded; and (8) full texts cannot be obtained or the data cannot be extracted.

### Identification of Eligible Trials

For search strategy, we searched articles published before December 2019 in the following databases: Chinese Science and Technology Periodical Database (VIP), China National Knowledge Infrastructure (CNKI), Wan Fang Database, PubMed, Embase, Web of Science, and the Ovid Library, and using the combining medical subject headings and keyword terms for stroke, acupuncture, and cognition. The search terms included “acupuncture/electroacupuncture” AND “stroke/stroke rehabilitation/cerebrovascular accident/brain ischemia/cerebral hemorrhage/CVA/cerebral embolism” AND “cognition/cognitive.” At the same time, some studies were extracted from the references in the full-text articles. Articles were restricted to English and Chinese languages.

### Assessment of Risk of Bias

The methodological quality and the risk of bias of the included studies were compiled using the risk of bias tool in the Cochrane Handbook for Systematic Reviews of Interventions (version 5.3) by two reviewers (L.Z. and Y.W.) independently. This instrument included seven specific domains: random sequence generation, allocation concealment, blinding of participants and personnel, blinding of outcome assessment, incomplete outcome data, selective reporting, and other bias.

### Data Extraction

Studies were screened by two investigators independently. Disagreements were settled by consensus or a third investigator. The extracted data included general characteristics (author and year of publication), patient characteristics (sample size, mean age, and disease type), intervention characteristics (type and duration), and main outcomes and adverse events. When a given study reported the outcome with more than one cognitive function assessment, we gave preference primarily to MMSE or MoCA.

### Statistical Analysis

All statistical analyses were performed with Revman 5.3 software (The Cochrane Collaboration software unpdate). Since the outcomes in studies were continuous variables, the mean differences (MDs) with 95% confidence intervals (CIs) were calculated. Heterogeneity was showed by *I*^2^ index values with a *p*-value and percentage, respectively. A fixed-effects model would be used in a meta-analysis when heterogeneity was adopted (*I*^2^ < 25% or 50% > *I*^2^ ≥ 25% with *p* > 0.1). Otherwise (*I*^2^ ≥ 50% or 50% > *I*^2^ ≥ 25% with *p* ≤ 0.1) the random-effects model would be used.

The stability of the results was confirmed by sensitivity analysis. Publication bias was assessed by Begg's test with STATA software (version 12.0, Stata Corp). Quality of evidence was assessed with GRADEpro in website (www.gradepro.org).

## Results

### Eligible Studies

**Figure 1:**
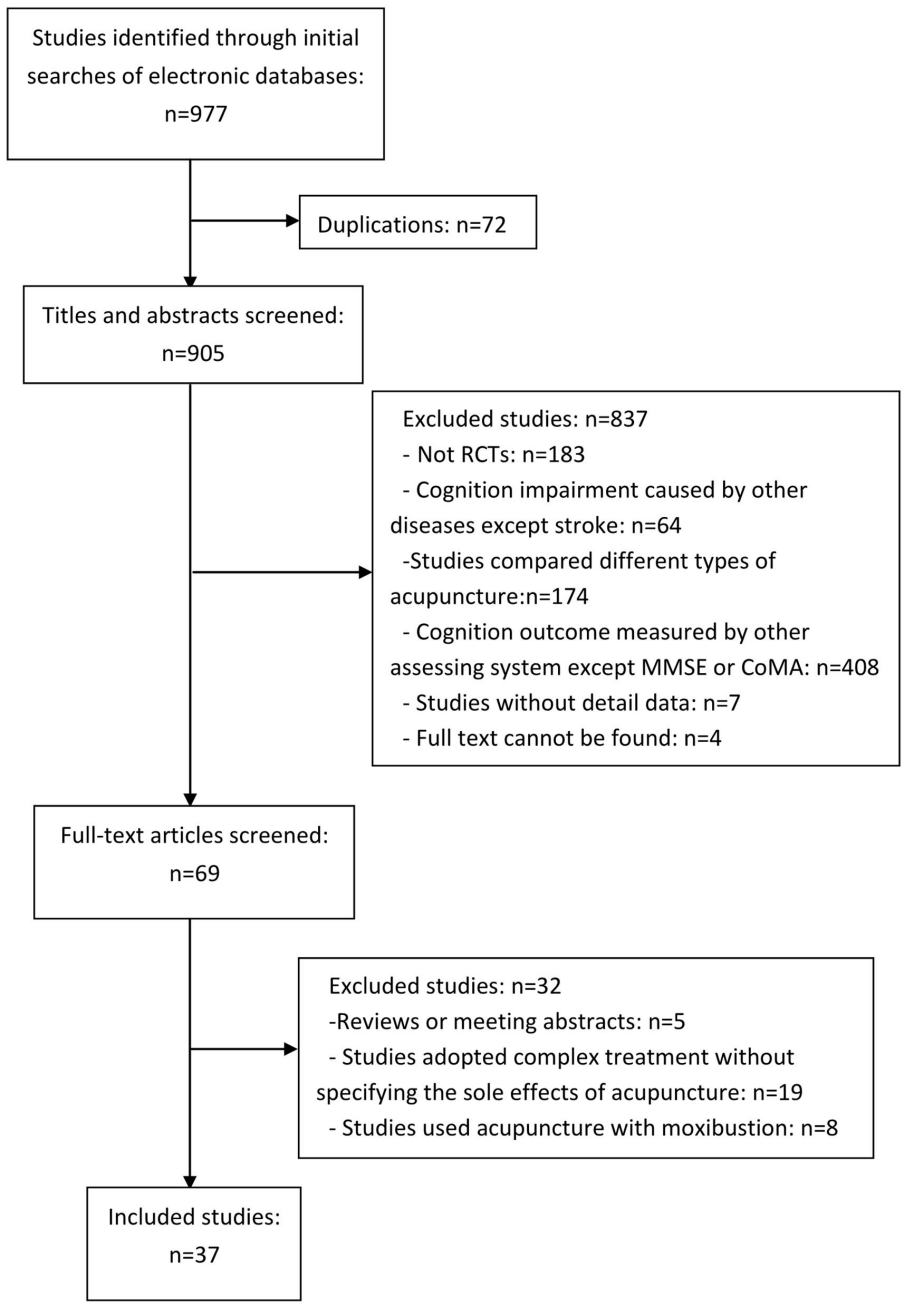
Literature screening flow diagram.

The workflow of literature screening and inclusion is shown in Figure 1. The initial literature search yielded 977 studies. Out of 977 studies, 72 studies were duplicated. A total of 905 studies were assessed for eligibility by titles and abstracts screening. There were 69 papers that compared the effect of acupuncture in patients with PSCI. With full-text reading, 32 articles were excluded, and 37 studies were included in the synthesis. The 37 studies are 31 journal articles (Huang et al., 2008; Li and Zhang, 2008; Lin et al., 2010; Jia and Meng, 2011; Sun and Wu, 2011; Bai et al., 2012; Li et al., 2012, 2019; Liu and Feng, 2013; Song et al., 2013; Wang, 2014, 2019; Wang et al., 2014, 2019; Yang, 2014; Liu et al., 2015a,b; Zeng et al., 2015; Cai et al., 2016; Shao, 2016; Liu, 2017; Wang H. et al., 2017; Wang Z. et al., 2017; Zhang et al., 2017; Du et al., 2018; Jia and Lv, 2018; Ma et al., 2018; Wang and Li, 2018; Shi and Wei, 2019; Zhou H. et al., 2019; Zhou J. et al., 2019) and 6 dissertations (Jiang, 2011; Kang, 2011; Yang, 2011; Feng, 2013; Lu, 2014; Sun, 2017), which involved 2,869 patients (1,442 patients in the treatment group and 1,427 patients in the control group) in total. All those studies were conducted in China. Thirty-six papers were published in the Chinese language. Table 1 shows the detailed information on the characteristics of the included studies. The ages of the patients range from 35 to 80 years. Seven trials did not describe the sex of the patients, while other trials included more male than female participants. The treatment period ranged from 2 to 12 weeks; the frequency of the sessions ranged from two sessions per day to five sessions per week. The chronicity of stroke ranged from 3 to 1,080 days, but most of those patients were treated within 6 months of onset. Twenty-three trials were conducted by manual acupuncture stimulation, and the other 14 trials used electroacupuncture only. The cognitive function assessment of all included studies was MMSE or MoCA.

**Table 1:** Characteristics of included studies.

**References**	**Patients no**.		**Ages (years)**		**Type of stroke**	**Outcome measures (MMSE/MoCA)**	**Therapy duration (wk)**	**Intervation**		**Source of diagnostic criteria for cerebral vascular diseases**	**Source of diagnostic criteria for PSCI**
	**Treatment**	**Control**	**Treatment**	**Control**				**Treatment**	**Control**		
Sun (2017)	30	28	60.63 ± 8.273	61.29 ± 7.693	Ischemic stroke or hemorrhage	MMSE and MoCA	6	Acupuncture+ control treatment	Conventional treatment + rehabilitation	FNACCVD confirmed by head CT or MRI	DSM-IV
Shao (2016)	28	28	63 ± 5		Ischemic stroke or hemorrhage	MMSE	12	Acupuncture+ control treatment	Conventional treatment + rehabilitation	CECS, Chinese expert consensus standards	DSM-IV
Liu et al. (2015a)	32	30	51.97 ± 9.11	51.30 ± 10.57	Ischemic stroke or hemorrhage	MoCA	4	Electropuncture+ control treatment	Conventional treatment + rehabilitation	CECS, Chinese expert consensus standards	MoCA
Cai et al. (2016)	52	49	57.75 ± 13.74	56.18 ± 11.86	Ischemic stroke or hemorrhage	MMSE and MoCA	12	Acupuncture+ control treatment	Conventional treatment + rehabilitation	CECS, Chinese expert consensus standards	MMSE
Zeng et al. (2015)	50	50	66 ± 12	68 ± 10	Ischemic stroke or hemorrhage	MoCA	4/8	Electropuncture+ control treatment	Conventional treatment + rehabilitation	FNACCVD confirmed by head CT or MRI	MoCA
Wang (2014)	33	31	66.4 ± 3.0		Ischemic stroke or hemorrhage	MMSE	3	Acupuncture+ control treatment	Conventional treatment + xingnaojing	FNACCVD confirmed by head CT or MRI	Not shown
Lu (2014)	30	30	63.27 ± 11.88	63.90 ± 8.48	Ischemic stroke or hemorrhage	MoCA	4	Acupuncture+ control treatment	Conventional treatment + rehabilitation	CECS, Chinese expert consensus standards	MoCA
Zhai (2012)	55	55	59.2		Ischemic stroke	MMSE	12	Acupuncture+ control treatment	Conventional treatment + rehabilitation	FNACCVD confirmed by head CT or MRI	Not shown
Bai et al. (2012)	30	30	60 ± 6	60 ± 6	Ischemic stroke or hemorrhage	MMSE	4	Acupuncture+ control treatment	Conventional treatment + piracetam	FNACCVD confirmed by head CT or MRI	CCSE
Li et al. (2012)	48	46	68.29 ± 8.22	69.22 ± 7.88	Ischemic stroke or hemorrhage	MMSE	12	Acupuncture+ control treatment	Conventional treatment + nimodipine	FNACCVD confirmed by head CT or MRI	MMSE
Yang (2011)	20	20	59.00 ± 8.46	59.30 ± 8.42	Ischemic stroke or hemorrhage	MMSE	8	Acupuncture+ control treatment	Conventional treatment + rehabilitation	FNACCVD confirmed by head CT or MRI	MMSE
Sun and Wu (2011)	36	36	63.6 ± 5.8	64.1 ± 5.5	Ischemic stroke	MMSE	4	Acupuncture+ control treatment	Conventional treatment + aricept	FNACCVD confirmed by head CT or MRI	MMSE
Kang (2011)	24	24	60.67 ± 6.93	62.71 ± 5.34	Ischemic stroke or hemorrhage	MMSE	8	Electropuncture+ control treatment	Conventional treatment + rehabilitation	FNACCVD confirmed by head CT or MRI	MMSE
Jiang (2011)	20	20	62.85 ± 5.67	61.75 ± 6.35	Ischemic stroke or hemorrhage	MMSE	8	Electropuncture+ control treatment	Conventional treatment + rehabilitation	FNACCVD confirmed by head CT or MRI	CCSE
Jia and Meng (2011)	50	50	65 ± 2	58 ± 3	Ischemic stroke	MoCA	12	Acupuncture+ control treatment	Conventional treatment + rehabilitation + nimodipine	CECS, Chinese expert consensus standards	Diagnosis criteria shown in reference (Jia, 2004)
Lin et al. (2010)	30	30	63 ± 17	56 ± 13	Ischemic stroke	MMSE	3	Acupuncture+ control treatment	Conventional treatment + xingnaojing	FNACCVD confirmed by head CT or MRI	MMSE
Huang et al. (2008)	40	40	59.22 ± 10.6	61.05 ± 9.68	Ischemic stroke	MMSE	4	Acupuncture+ control treatment	Conventional treatment + xingnaojing	FNACCVD confirmed by head CT or MRI	CECVCI
Shi and Wei (2019)	55	55	60.31 ± 2.73	60.24 ± 2.65	Stroke	MMSE	4	Acupuncture+ control treatment	Conventional treatment + rehabilitation	Not shown	Not shown
Zhou J. et al. (2019)	60	60	61.44 ± 8.77	62.04 ± 8.69	Ischemic stroke or hemorrhage	MMSE and MoCA	4	Electropuncture+ control treatment	Conventional treatment + rehabilitation	CECS, Chinese expert consensus standards	DSM
Feng (2013)	40	40	51.65 ± 12.47	52.13 ± 12.77	Ischemic stroke or hemorrhage	MMSE and MoCA	4	Electropuncture+ control treatment	Conventional treatment + rehabilitation	FNACCVD confirmed by head CT or MRI	DSM-IV
Wang et al. (2019)	59	59	68.88 ± 3.64	67.71 ± 3.02	Ischemic stroke	MMSE	4	Acupuncture+ control treatment	Conventional treatment + Atorvastatin	FNACCVD confirmed by head CT or MRI	There are symptoms such as memory loss
Li et al. (2019)	40	40	66.9 ± 5.9	67.4 ± 6.1	Ischemic stroke or hemorrhage	MMSE and MoCA	6/12	Acupuncture+ control treatment	Conventional treatment+ Donepezil	FNACCVD confirmed by head CT or MRI	DSM-IV-R
Zhang et al. (2017)	42	42	62.28 ± 10.68	63.07 ± 10.59	Stroke	MMSE	4	Acupuncture+ control treatment	Conventional treatment + rehabilitation + Atorvastatin	FNACCVD confirmed by head CT or MRI	There are symptoms such as memory loss
Wang H. et al. (2017)	30	30	53.27 ± 11.62	56.73 ± 9.31	Ischemic stroke or hemorrhage	MMSE	8	Electropuncture+ control treatment	Conventional treatment + rehabilitation	FNACCVD confirmed by head CT or MRI	DSM-IV
Zhou H. et al. (2019)	40	40	61.5 ± 5.7	61.5 ± 4.4	Ischemic stroke	MMSE and MoCA	6	Electropuncture+ control treatment	Conventional treatment + rehabilitation + Perindopril	FNACCVD confirmed by head CT or MRI	Not shown
Wang Z. et al. (2017)	30	30	61.13 ± 11.42	60.06 ± 11.17	Ischemic stroke or hemorrhage	MMSE and MoCA	8	Acupuncture+ control treatment	Conventional treatment + rehabilitation	FNACCVD confirmed by head CT or MRI	DSM-IV-R
Wang et al. (2019)	78	78	69.04 ± 3.48	68.92 ± 3.65	stroke	MMSE	4	acupuncture+ control treatment	Conventional treatment + Tongluofuzheng decoction	Not shown	Not shown
Wang and Li (2018)	64	64	71.42 ± 8.67	69.33 ± 7.56	Ischemic stroke	MMSE and MoCA	6/10	Acupuncture+ control treatment	Conventional treatment + rehabilitation + nimodipine	CECS, Chinese expert consensus standards	Diagnosis criteria shown in reference (Zhang and Wang, 2004)
Yang (2014)	40	40	61.7 ± 4.8		Stroke	MMSE	3	Acupuncture+ control treatment	Conventional treatment + xingnaojing	FNACCVD confirmed by head CT or MRI	Not shown
Ma et al. (2018)	30	30	60.97 ± 7.15	60.17 ± 6.56	Ischemic stroke or hemorrhage	MMSE	2/4	Electropuncture+ control treatment	Conventional treatment + Oxiracetam + hyperbaric oxygen therapy	FNACCVD confirmed by head CT or MRI	MMSE
Jia and Lv (2018)	40	39	58.33 ± 11.13	57.45 ± 12.37	Ischemic stroke or hemorrhage	MMSE	4	Acupuncture+ control treatment	Conventional treatment + Huoxuetongluo decoction	FNACCVD confirmed by head CT or MRI	CCSE
Liu (2017)	32	32	56.9 ± 10.3	56.4 ± 10.1	Ischemic stroke or hemorrhage	MMSE	2	Electropuncture+ control treatment	Conventional treatment + rehabilitation	FNACCVD confirmed by head CT or MRI	Not shown
Liu et al. (2015b)	19	16	52.42 ± 7.62	51.06 ± 11.62	Ischemic stroke or hemorrhage	MMSE and MoCA	4	Electropuncture+ control treatment	Conventional treatment + rehabilitation	CECS, Chinese expert consensus standards	MMSE
Sun et al. (2013)	60	60	62.50 ± 4.52	63.01 ± 4.67	Ischemic stroke	MMSE	4	Electropuncture+ control treatment	Conventional treatment + rehabilitation + nimodipine	FNACCVD confirmed by head CT or MRI	MMSE
Liu and Feng (2013)	25	25	53.40 ± 8.48		Ischemic stroke or hemorrhage	MMSE	4	Electropuncture+ control treatment	Conventional treatment + rehabilitation	FNACCVD confirmed by head CT or MRI	DSM-IV-R
Wang et al. (2014)	30	30	45~80		Ischemic stroke	MoCA	12	Acupuncture+ control treatment	Conventional treatment + nimodipine	FNACCVD confirmed by head CT or MRI	Not shown
Li and Zhang (2008)	20	20	58~76		Ischemic stroke	MMSE	4	Electropuncture+ control treatment	Conventional treatment + rehabilitation	FNACCVD confirmed by head CT	Not shown

*MMSE, Mini-Mental State Examination; MoCA, Montreal Cognitive Assessment; FNACCVD, Fourth National Academic Conference of Cerebral Vascular Diseases; CT, computed tomography; MRI, magnetic resonance imaging; DSM-IV,Diagnostic and Statistical Manual of Mental Disorders(the fourth edition); DSM-IV-R, DSM-IV-Revised edition; CECS, Chinese expert consensus standards, proposed in 2005 for the prevention and treatment of cognitive dysfunction; CCSE, Cognitive Capacity Screening Examination; CECVCI, Chinese Expert consensus on vascular cognitive impairment 2007*.

### Assessment of Risk of Bias

All RCTs had a low risk of bias (ROB) about adequate sequence generation. Eight RCTs had a low ROB with allocation concealment, while 9 RCTs had a high ROB, and 20 had an unclear ROB. Concerning participant blinding, one RCT had low ROB and the others had a high or unclear ROB. About assessor blinding, only three RCTs had a low ROB.

**Figure 2:**
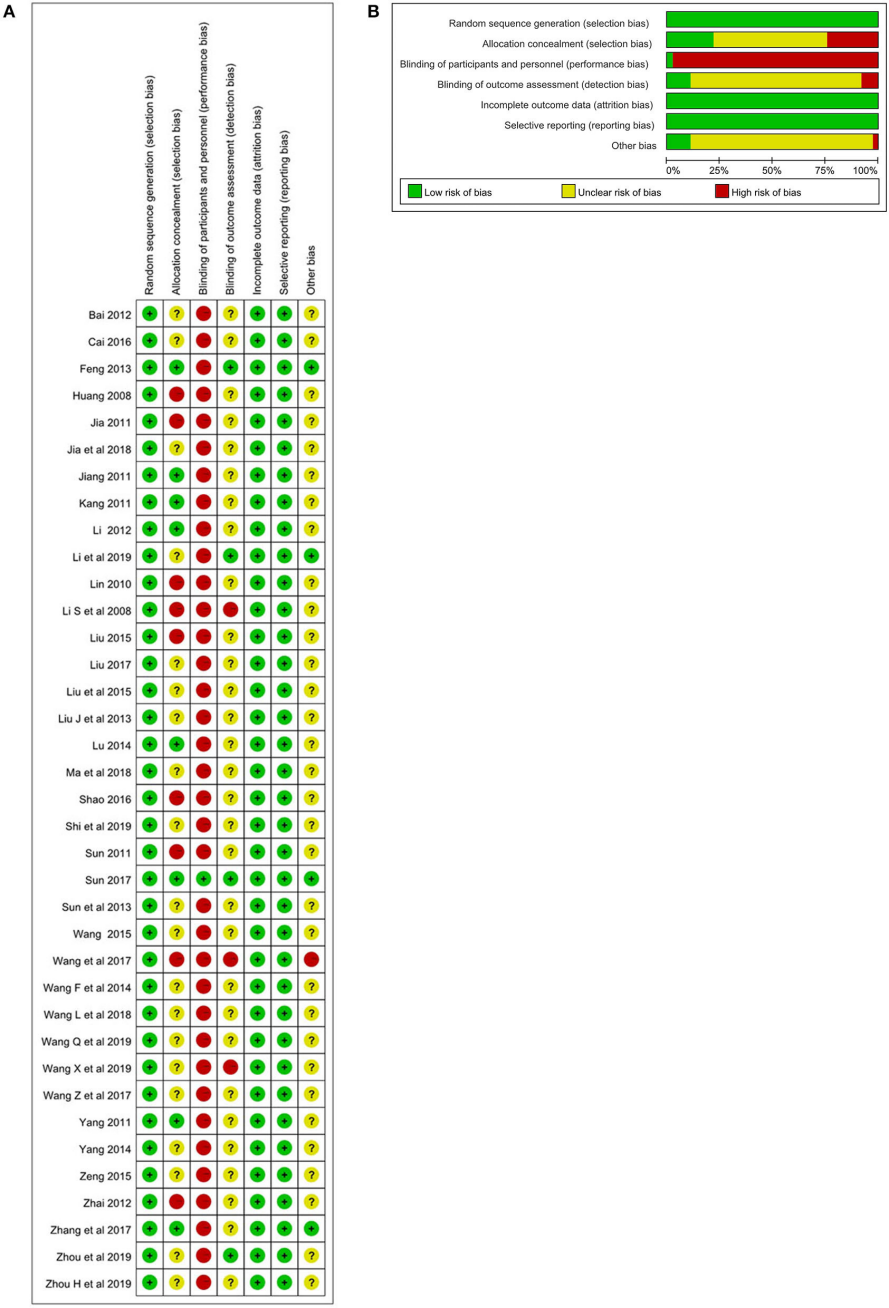
**(A)** Assessment of risk of bias with selected studies. **(B)**. Risk of bias graph and summary.

All 37 RCTs had a low ROB in incomplete outcome data addressed and selective outcome reporting. Thirty-four RCTs had an unclear ROB in other sources of bias. The results of the ROB assessment are shown in Table 2, Figure 2.

**Table 2:** Quality assessment of studies.

**References**	**Adequate sequence generation**	**Allocation concealment**	**Blinding of participation and personnel**	**Blinding of outcome assessment**	**Incomplete outcome data addressed**	**Selective outcome reporting avoided**	**Other sources of bias**
Sun (2017)	Yes	Yes	Yes	Yes	Yes	Yes	Unclear
Shao (2016)	Yes	No	No	Unclear	Yes	Yes	Unclear
Liu et al. (2015a)	Yes	No	No	Unclear	Yes	Yes	Unclear
Cai et al. (2016)	Yes	Unclear	No	Unclear	Yes	Yes	Unclear
Zeng et al. (2015)	Yes	Unclear	No	Unclear	Yes	Yes	Unclear
Wang (2014)	Yes	Unclear	No	Unclear	Yes	Yes	Unclear
Lu (2014)	Yes	Yes	No	Unclear	Yes	Yes	Unclear
Zhai (2012)	Yes	No	No	Unclear	Yes	Yes	Unclear
Bai et al. (2012)	Yes	Unclear	No	Unclear	Yes	Yes	Unclear
Li et al. (2012)	Yes	Yes	No	Unclear	Yes	Yes	Unclear
Yang (2011)	Yes	Yes	No	Unclear	Yes	Yes	Unclear
Sun and Wu (2011)	Yes	No	No	Unclear	Yes	Yes	Unclear
Kang (2011)	Yes	Yes	No	Unclear	Yes	Yes	Unclear
Jiang (2011)	Yes	Yes	No	Unclear	Yes	Yes	Unclear
Jia and Meng (2011)	Yes	No	No	Unclear	Yes	Yes	Unclear
Lin et al. (2010)	Yes	No	No	Unclear	Yes	Yes	Unclear
Huang et al. (2008)	Yes	No	No	Unclear	Yes	Yes	Unclear
Shi and Wei (2019)	Yes	Unclear	No	Unclear	Yes	Yes	Unclear
Zhou J. et al. (2019)	Yes	Unclear	No	Unclear	Yes	Yes	Unclear
Feng (2013)	Yes	Yes	No	Yes	Yes	Yes	No
Wang et al. (2019)	Yes	Unclear	No	Unclear	Yes	Yes	Unclear
Li et al. (2019)	Yes	Unclear	No	Yes	Yes	Yes	No
Zhang et al. (2017)	Yes	Yes	No	Unclear	Yes	Yes	No
Wang H. et al. (2017)	Yes	No	No	No	Yes	Yes	Unclear
Zhou H. et al. (2019)	Yes	Unclear	No	Unclear	Yes	Yes	Unclear
Wang Z. et al. (2017)	Yes	Unclear	No	Unclear	Yes	Yes	Unclear
Wang et al. (2019)	Yes	Unclear	No	Unclear	Yes	Yes	Unclear
Wang and Li (2018)	Yes	Unclear	No	Unclear	Yes	Yes	Unclear
Yang (2014)	Yes	Unclear	No	Unclear	Yes	Yes	Unclear
Ma et al. (2018)	Yes	Unclear	No	Unclear	Yes	Yes	Unclear
Jia and Lv (2018)	Yes	Unclear	No	Unclear	Yes	Yes	Unclear
Liu (2017)	Yes	Unclear	No	Unclear	Yes	Yes	Unclear
Liu et al. (2015b)	Yes	Unclear	No	Unclear	Yes	Yes	Unclear
Sun et al. (2013)	Yes	Unclear	No	Unclear	Yes	Yes	Unclear
Liu and Feng (2013)	Yes	Unclear	No	Unclear	Yes	Yes	Unclear
Wang et al. (2014)	Yes	Unclear	No	Unclear	Yes	Yes	Unclear
Li and Zhang (2008)	Yes	No	No	No	Yes	Yes	Unclear

### Meta-Analysis of the Results

**Figure 3:**
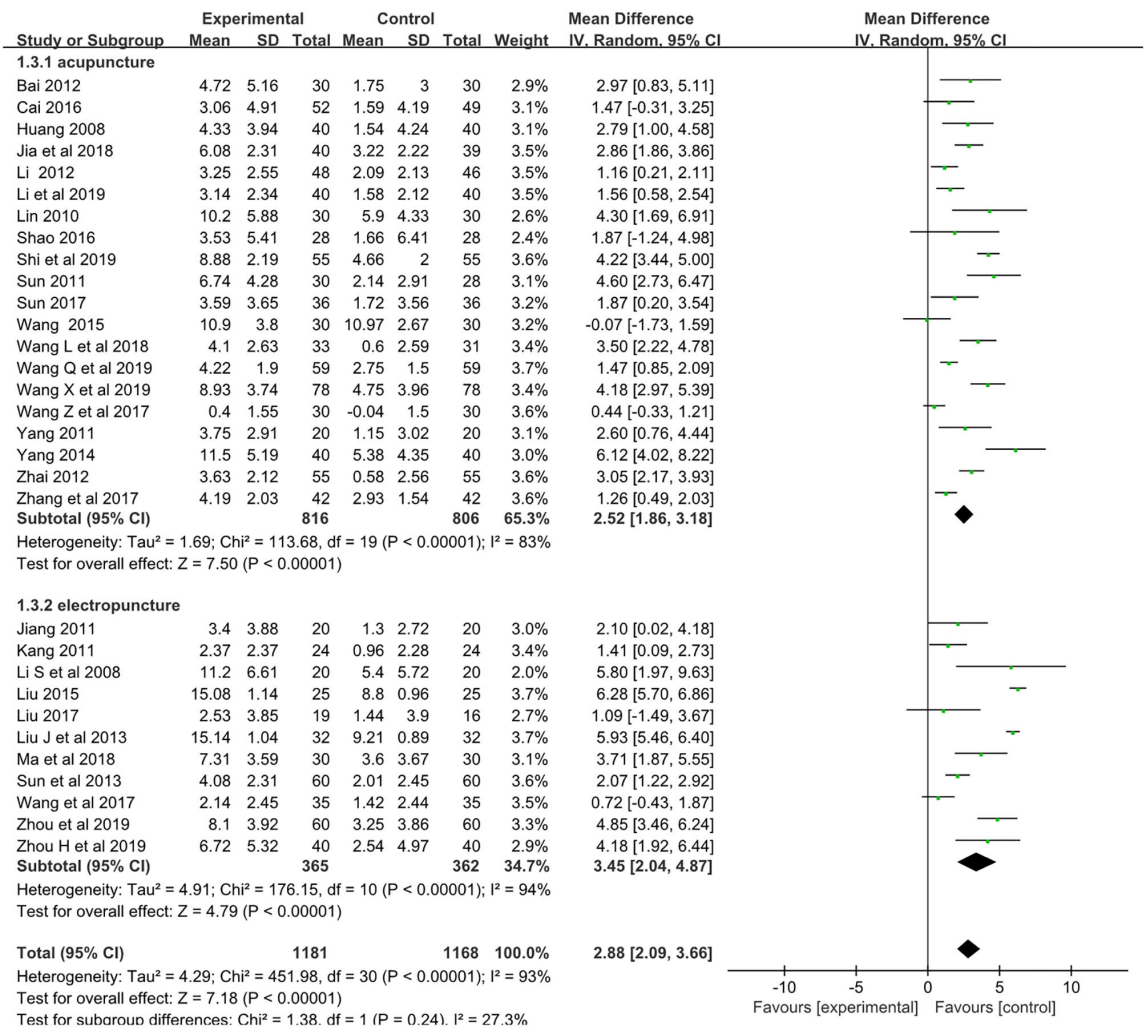
Forest plot comparing the MMSE improved between acupuncture groups and control groups.

The pooled meta-analysis of the data showed a weighted mean difference of 2.88 and 95% confidence intervals (CI) of 2.09–3.66 on the MMES (*p* < 0.001, *n* = 2,349; Figure 3).

Subgroup analyses showed weighted mean differences of 2.52 (95% CI: 1.86–3.18, *n* = 1,622) and 3.45 (95% CI: 2.09–3.66, *n* = 727) for acupuncture subgroup and electropuncture subgroup, respectively.

**Figure 4:**
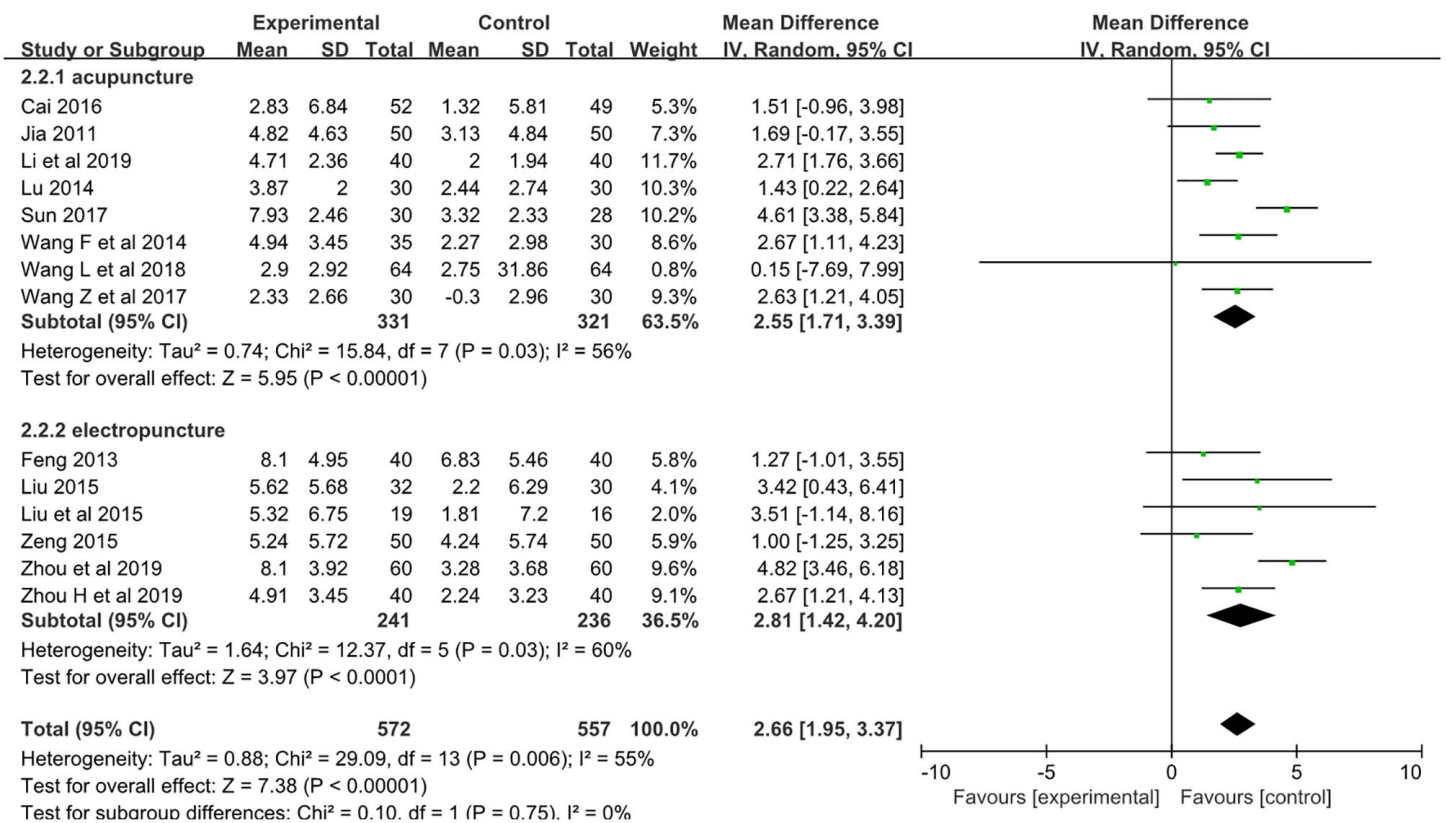
Forest plot comparing the CoMA improved between acupuncture groups and control groups.

The pooled meta-analysis of the data showed a weighted mean difference of 2.66 and 95% confidence intervals of 1.95–3.37 on the MoCA (*p* < 0.001, *n* = 1,129; Figure 4).

Subgroup analyses showed weighted mean differences of 2.55 (95% CI: 1.71–3.39, *n* = 652) and 2.81 (95% CI: 1.42–4.02, *n* = 477) for acupuncture subgroup and electropuncture subgroup, respectively.

The results indicated that acupuncture had a significant effect on PSCI, and no adverse events were reported in those studies.

### Sensitivity Analysis, Publication Bias, and Overall Quality of Evidence

**Figure 5:**
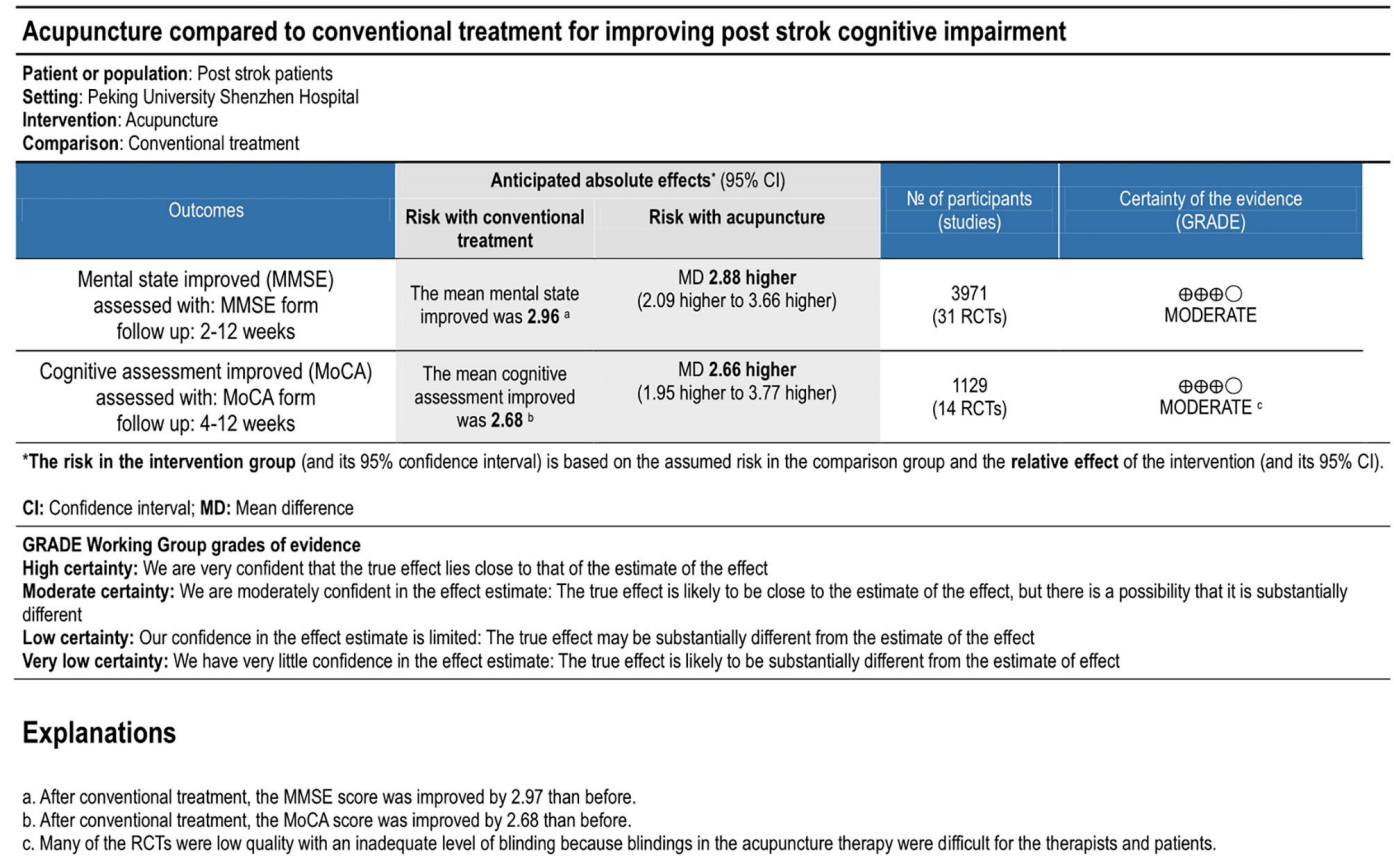
Evidence profile.

High heterogeneity was shown in results (*I*^2^ = 93 and 55 for MMSE and MoCA, respectively), so subgroup analyses were done based on different methods of acupuncture between manual acupuncture (acupuncture) and electropuncture. The results of subgroup analyses showed that intra-group heterogeneity remained high in subgroups (*I*^2^ > 50), and the inter-group heterogeneity between subgroups was not too much (*I*^2^ < 50). Then sensitivity analysis was conducted by excluding the maximum weight studies in outcomes on subgroup analyses. The results showed that there was little influence on the pooled MD value. Then a study was removed at a time and the others analyzed to assess whether the results could have been influenced significantly by a single study. The results also showed no apparent fluctuation. These analyses confirmed the stability of the results of pooled MD value. Begg's tests showed no significant publication bias with symmetrical funnel plots. The overall quality of evidence was rated as moderate for MMSE and MoCA (Figure 5).

## Discussion

Our findings showed that acupuncture or electroacupuncture therapy is effective in improving the cognitive impairment of post-stroke patients by assessing with MMSE and MoCA. The gain of the mean difference is 2.88 for MMSE (CI [2.09, 3.66]), which is significant in clinical treatment (Andrews et al., 2019). The gain of the mean difference is 2.66 for MoCA (CI [1.95, 3.37]), which is also significant in clinical treatment (Wong et al., 2017).

In this study, patients in the control group were treated with conventional treatment in all 37 trials, patients had conventional rehabilitation done in the control groups in 23 trials, and patients had medicine in control groups in 17 trials. Patients in experiment groups combined the acupuncture or electroacupuncture and conventional rehabilitation or medicine used the same as in control groups in all trials. The merged results showed that synergistic effects of acupuncture or electroacupuncture therapy is clinically significant in improving PSCI, and there were no adverse events/incidents reported in those studies.

There was some inconsistent information in the included studies. The patients were all with ischemic stroke in 10 studies; the other 22 studies included patients with hemorrhage or ischemic stroke, and 4 studies only included post-stroke patients. In the 37 studies, 23 studies indicated that acupuncture treatment was within 6 months from stroke onset, 5 studies was under 1 year, 2 studies was under 14/36 months, and the other 7 studies did not report the accurate time. In this meta-analysis, 24 studies focused on the effects of acupuncture combined with conventional rehabilitation treatment, and the other 13 studies analyzed the effectiveness of acupuncture combined with medicine (Aricept, Xingnaojing, Nimodipine, Piracetam, etc.). Fourteen studies used electroacupuncture, and the other 23 studies used traditional manual acupuncture. The intervention period varied across studies from 2 to 12 weeks.

There were obvious heterogeneities of these articles, so the random effects model was used in this study. Subgroup analysis between acupuncture group and electropuncture did not significantly reduce heterogeneity in this study. This may be caused by the unbalance of acupoints selection, the different treatment period, and the therapist's technical ability.

In the theoretical system of acupuncture, the Du Meridian is important for the cognitive brain function (Zhou et al., 2013; Liu et al., 2014), and acupoints “Baihui” and “Shenting” belong to the Du Meridian. Baihui and Shenting are both located in the head. To Chinese traditional medicine theoretical system, acupuncture Baihui and Shenting can lift the spirit, clear the mind, and promote resuscitation. In these 37 studies, 26 studies acupuncture the acupoints including “Baihui,” and 19 studies involved the acupoint “Shenting” for the treatment of mental and emotional illness. Other acupoints, such as Feishu, Xinshu, Ganshu, Shenshu, and Pishu were shown involving the cognitive function in more than three studies. Other acupuncture points involved were Huiyin, Yintang, Neiguan, Yanglingquan, Taixi, Zulinqi, Sishencong, Fengchi, Fengfu, Gongxue, Yiming, Guanyuan, Taichong, Shenshu, Benshen, Hegu, Taichong, Fengshi, Quchi, Zusanli, Sanyingjiao, Xuehai, Renzhong, Shenmen, etc.

Acupuncture improves cognitive function and depressive disorder, because acupuncture on stroke patients can improve neurological function (Chen et al., 2018; Hung et al., 2019). Animal studies showed that acupuncture with Baihui may have a neuroprotective effect via decreasing MMP-9 expression or improving the endothelial nitric oxide synthase (eNOS)-mediated perfusion (Dong et al., 2009; Kim et al., 2013).

H Jiang et al.'s and J Liang et al.'s studies showed that acupuncture was associated with the potential of DNA methylation and histone modifications of brain-derived neurotropic factor in epigenetic mechanism, which can produce antidepressant effect in rats (Liang et al., 2012; Jiang et al., 2018). F Taya et al. showed that acupuncture may increase cerebral collateral circulation, promoting repair of the lesion (Taya et al., 2015). P.Y. Sun et al. showed that acupuncture repairs hippocampal neuronal damage, which is probably related to the contents of hippocampal monoamine neurotransmitters (NE, 5-HT and DA) (Sun et al., 2019). Other studies showed that electropuncture can improve cognitive function via synaptic plasticity by attenuating pathological lesions and increasing the density of dendritic spines and number of CA1 synapses in rats (Lin et al., 2016; Liu et al., 2017; Wen et al., 2018).

The selection criteria for the assessment of cognitive function were the Mini-Mental State Examination (MMSE) and the Montreal Cognitive Assessment (MoCA). MMSE is an effective tool that can be used to systematically and thoroughly assess mental status, which was validated and extensively used from 1975 (Foreman et al., 1996). MoCA is a widely used screening assessment for detecting cognitive impairment since 1996, which was validated in the setting of mild cognitive impairment (Nasreddine et al., 2005). There are other internationally recognized examinations of cognitive impairment including NCSE, NIHSS, LOTCA, HDS, and cognitive potential 300, but the most commonly used indicators are MMSE and MoCA. We restricted the inclusion criteria to a consistent standard of outcome assessing with MMSE or MoCA, so the number of RCTs included in this study was not so many (only 37), but the results of meta-analyses were more clear and definite, and the quality of evidence was assessed to be moderate.

About the limitation, firstly, all studies were done in China, although the Cai et al. (2016) study was published in the English language. There might have been additional reports using non-Chinese or non-English languages that were not included which may limit the results of the study. Secondly, many of the trials were of low quality with an inadequate level of blinding; although blinding in the acupuncture therapy is difficult for the therapists and patients, blinding the assessor is necessary.

Despite these limitations, conclusions can be drawn from the results of our study.

## Conclusions

Acupuncture therapy has positive synergistic effects in improving PSCI, but more rigorous design studies with large-scale sham are needed to determine the longevity of acupuncture effects.

## Data Availability Statement

The original contributions presented in the study are included in the article/supplementary materials, further inquiries can be directed to the corresponding author/s.

## Author Contributions

LZ, QW, and XL: conceptualization and writing, review, and editing. LZ, YW, and JQ: data curation and methodology. LZ and XL: funding acquisition. QW and XL: supervision. LZ: writing the original draft. All authors contributed to the article and approved the submitted version.

## Conflict of Interest

The authors declare that the research was conducted in the absence of any commercial or financial relationships that could be construed as a potential conflict of interest.
